# Psychosocial work environment as a dynamic network: a multi-wave cohort study

**DOI:** 10.1038/s41598-022-17283-z

**Published:** 2022-07-28

**Authors:** Marko Elovainio, Christian Hakulinen, Kaisla Komulainen, Mika Kivimäki, Marianna Virtanen, Jenni Ervasti, Tuula Oksanen

**Affiliations:** 1 Research Program Unit, Faculty of Medicine, University of Helsinki, P.O. Box 9, Helsinki, Finland; 2 Finnish Institute for Health and Welfare, Helsinki, Finland; 3 Department of Psychology and Logopedics, University of Helsinki, Helsinki, Finland; 4 Finnish Institute of Occupational Health, Helsinki, Finland; 5 Department of Epidemiology and Public Health, University College London, London, UK; 6 Clinicum, Faculty of Medicine, University of Helsinki, Helsinki, Finland; 7 School of Educational Sciences and Psychology, University of Eastern Finland, Joensuu, Finland; 8 Institute of Public Health and Clinical Nutrition, University of Eastern Finland, Kuopio, Finland

**Keywords:** Psychology, Human behaviour

## Abstract

While characteristics of psychosocial work environment have traditionally been studied separately, we propose an alternative approach that treats psychosocial factors as interacting elements in networks where they all potentially affect each other. In this network analysis, we used data from a prospective occupational cohort including 10,892 participants (85% women; mean age 47 years) and repeated measurements of seven psychosocial work characteristics (job demands, job control, job uncertainty, team climate, effort-reward imbalance, procedural justice and interactional justice) assessed in 2000, 2004, 2008 and 2012. Results from multilevel longitudinal vector autoregressive models indicated that job demands as well as interactional and procedural justice were most broadly associated with the subsequent perceptions of the work-related psychosocial factors (high out-Strength), suggesting these factors might be potentially efficient targets of workplace interventions. The results also suggest that modifying almost any of the studied psychosocial factors might be relevant to subsequent perceptions of effort-reward imbalance and interactional justice at the workplace.

## Introduction

People spend a large share of their waking hours at work. Several systematic reviews and meta-analyses have shown that characteristics of the psychosocial work environment are important for overall wellbeing^1–5^. The psychosocial work characteristics that have often been studied include, for example, time pressure and work overload, too difficult or demanding tasks, poor job control, poor social support or social relations, social isolation, uncertainty, injustice, role ambiguity or role conflicts, poor climate or team work, work-family interference, effort-reward imbalance and monotonic or repetitive tasks, poor equipment and high responsibility^6–24^. A central motivation for identifying, measuring, and understanding the relevant aspects of psychosocial work environment is to be able to effectively improve work life and eventually employee well-being. As described above, previous research has identified many individual work-related psychosocial factors potentially affecting employee well-being, traditionally focusing on one or a few of these factors.

In this paper, we will build on an idea that psychosocial work environment is not a collection of a few independent characteristics, but a dynamic system comprising multiple interconnected psychosocial components^25^. In this perspective, each individual psychosocial factor is a part of a unique ‘system of temporal associations’, that is, a pattern of potential causes and effects that each psychosocial factor exhibits in relation to other factors. We hypothesize that such factors are unlikely to change independently of one another—instead, they may form a network of mutual dependencies—and that a key feature of a given psychosocial environment is the architecture and density of connections between individual psychosocial factors forming systemic states. For example, high workload may affect a person’s ability to control their own work, thus influencing perceptions of decision-making latitude, which may potentially lead to a generalized experience of unfairness, which not only affects the perception of control but also other psychosocial factors, such as trust and job certainty.

Thus, we will apply a psychological network approach^26,27^ in the analysis of psychosocial work characteristics. The network approach has recently been utilized in studies on mental health, and this line of research is rapidly expanding^27–30^. There are two main reasons for the rapid growth: (1) recent developments in statistical methods provide opportunities to analyse dynamic weighted networks (i.e. incorporate information on the magnitude and direction of network associations over time)^31–33^, and (2) recently constructed theoretical frameworks have developed our understanding of psychiatric symptoms^34–36^, personality traits^37^, health measures^29^ and attitudes^38^, and even psychosocial risks^25^ as potential network structures. The network framework is generally focused on individual factors that have unique sets of interrelated associations with other factors within a network. These associations can be visually illustrated as networks comprising nodes (factors of interest) and edges (associations between the factors). Furthermore, the network approach allows for examining the centrality of each factor in the overall architecture of the network.

Research on psychosocial work characteristics has traditionally been based on specific theoretical models, which typically focus on a limited set of specific psychosocial characteristics. More general approaches of work related issues involve a wider set of factors, such as the Motivation Potential -model^39^ and the Conservation of Resource -model^40^ while other models focus on more specific aspects of work and social relations, such as the Job Strain model^7^, the Effort-Reward Imbalance model (ERI)^20^, the Team Climate Model^41^, the Uncertainty Model^42^ and the Organizational Justice model^24^. These models have all been rigorously tested and all of them have gained empirical support in predicting health and wellbeing. These models have generally been understood as complementary, each concentrating on different aspects of work environment. The Job Strain Model^7,12^, also known as the Demand–Control Model, proposes that employees working under high strain (a combination of high work demands and low job control) have a higher risk of reduced wellbeing and more health problems than those without such strain^43^. As an alternative conceptualization, the Uncertainty model suggests that work-related factors associated with uncertainty, such as the threat of job loss, are among the most stressful situations threatening multiple resources and affecting social relations at work^44–46^. The Team Climate Model, in turn, is focused on employees’ shared perceptions and interpretations of the organizational environment, especially factors related to co-operation, such as safe participation, shared goals and mutual trust.

Several psychosocial models are based on the fairness principle. The Effort-Reward Imbalance model, for example, is focused on two work characteristics, high effort, and lack of rewards, as indicators of stressful working conditions. Efforts refer to the demands and obligations the employee is faced with, and rewards to the monetary rewards, esteem, and career opportunities the employee subsequently expects. Asymmetry in the exchange of efforts and rewards, that is, high efforts combined with low rewards, is hypothesized to cause emotional distress and elevated autonomic arousal that, in turn, may cause health problems. The Organizational Justice Model, in turn, is focused on the extent employees are treated with justice at their workplace (for a review see^47^). Organizational justice has been shown to predict organizational attitudes, such as commitment and involvement^48^. There is also a large body of evidence supporting a link between unfair treatment, experienced strain and various health problems^23,24,49–51^.

Each of these models include only a limited set of dimensions of the psychosocial work environment, partly because they have relatively specific theoretical frameworks developed in particular historical settings to describe health-related aspects of the evolving work life. Although all of them cover some important parts of the psychosocial work environment, the psychosocial factors proposed by these models have rarely been assessed within a single analytic framework or within one study population. Even in the most comprehensive studies^52–54^, the specific psychosocial factors have been treated as additive and unrelated. Recently, more encompassing theoretical models on psychosocial work environment and their potential overlap have been presented, but again based on evidence from studies evaluating individual dimensions separately^55,56^.

Although the most recent research based on the Network approach focuses on mental disorders, the approach could also be applied to several other phenomena, including psychosocial risks and resources^25^. As the Network approach does not assume a latent structure underlying the associations between various lower-level (item-level justice or job control) or higher-level (procedural justice or job control as theoretical constructs) psychosocial factors, it could offer an integrated explanation for the interconnectedness of work-related psychosocial factors (architecture or risks and resources), as well as inform about which factors are particularly *central* in activating multiple other factors^57^. Identifying the most central factors could provide information about the useful targets of psychosocial interventions, given that a reduction in the most central risks could be expected to reduce multiple other risks within the work environment. Similarly, strengthening some central psychosocial resources might also enhance other key resources.

Here, we utilize the network approach to evaluate the interconnections between work-related psychosocial risks and resources in a prospective cohort study among over 10 000 participants, who provided repeated-measures data on several work-related psychosocial factors at four measurement points over 12 years. Mapping the structure of psychosocial work environment onto a network is a challenging task. We start with the most established psychosocial components that could be plausible candidates to make up a work-related psychosocial environment network structure.

## Results

We included seven psychosocial factors from five widely tested models: the Job Strain Model (job control and job demands as separate psychosocial factors), the Team Climate Model, the Job Uncertainty Model, the Effort-Reward imbalance Model, and the Organizational Justice Model (procedural and interactional justice as separate psychosocial factors). These seven psychosocial factors were included in all network analyses. Following the procedure suggested by Jongeneel and others^58^, we calculated the within-individual means and standard deviations, as well as the ranges of intra-individual means and intra-individual standard deviations for all psychosocial factors across the measurement points (Table 1).

**Table 1:** Mean, standard deviation, and range of intra-individual means and intra-individual standard deviations per variable.

Psychosocial factor	Mean	SD of mean	Min of mean	Max of mean	SD	SD of SD	Min of SD	Max of SD
Procedural justice	3.71	0.67	1.00	5.00	0.65	0.37	0.00	2.31
Interactional justice	3.08	0.66	1.00	5.00	0.57	0.31	0.00	2.23
ERI	1.54	0.38	0.49	4.38	0.32	0.25	0.00	2.00
Job demands	3.19	0.70	1.00	5.00	0.56	0.28	0.00	1.85
Job control	3.73	0.53	1.33	5.00	0.30	0.17	0.00	1.73
Team Climate	3.56	0.52	1.21	4.98	0.40	0.22	0.00	1.88
Uncertainty	2.07	0.53	1.00	5.00	0.48	0.27	0.00	1.91

Year	Procedural justice	Interactional justice	Effort-reward imbalance	Job demands	Uncartainty	Job control	Team climate	
**Crombach's alphas of the psychosocial factors measured in years 2000–2012**								
2000	0.92	0.91	0.64	0.77	0.51	0.82	0.92	
2004	0.93	0.91	0.65	0.77	0.52	0.81	0.92	
2008	0.93	0.92	0.68	0.77	0.52	0.82	0.93	
2012	0.93	0.91	0.68	0.77	0.55	0.81	0.92	

All variables (except ERI) has a potential score range from 1 to 5.

N = 10,892.

**Figure 1:**
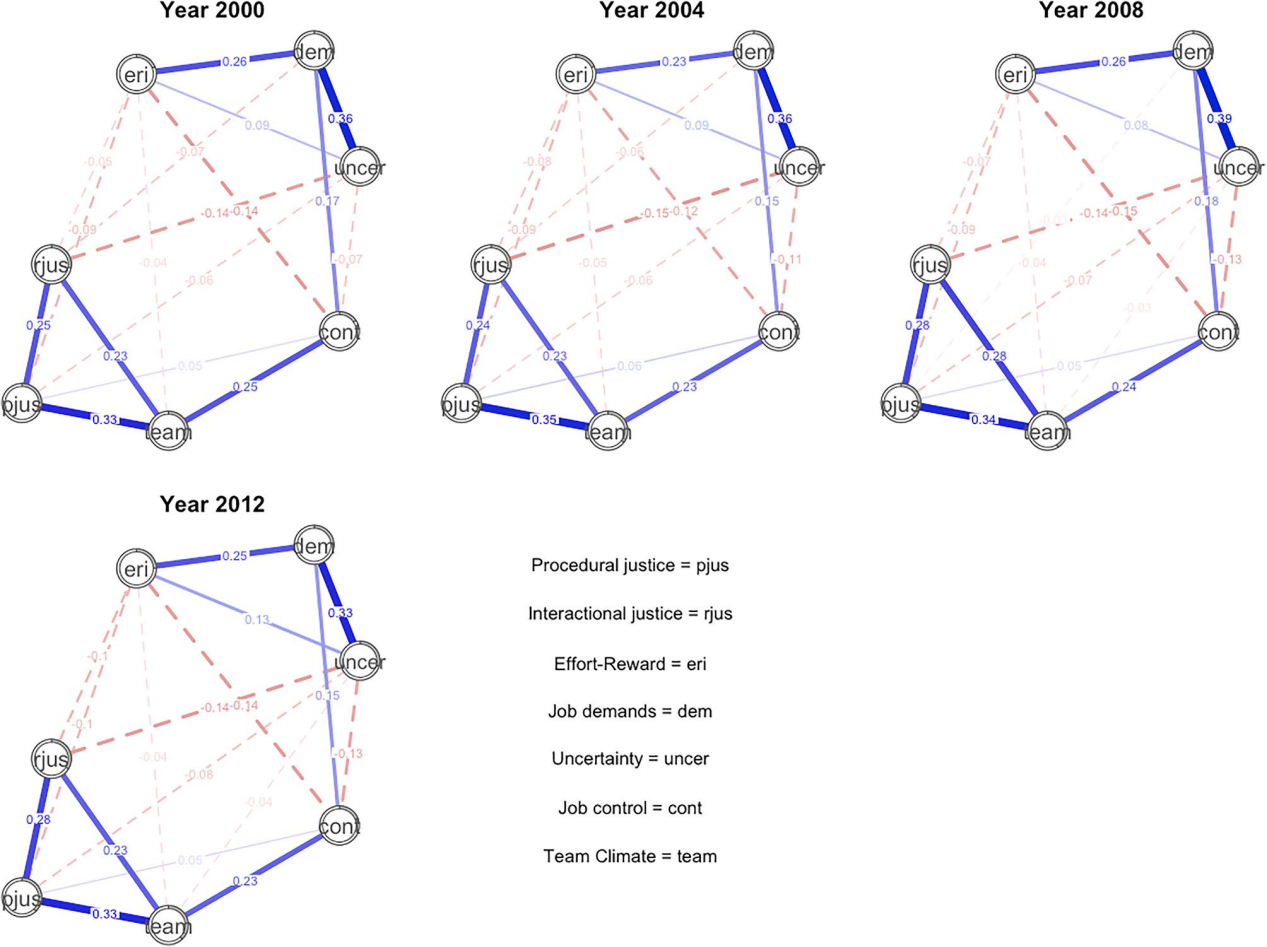
Cross-sectional networks for years 2000, 2004, 2008 and 2012 (procedural justice = pjus, interactional justice = rjus, effort-reward imbalance = eri, job demands = dem, job control = cont, uncertainty = uncer, team climate = team).

**Figure 2:**
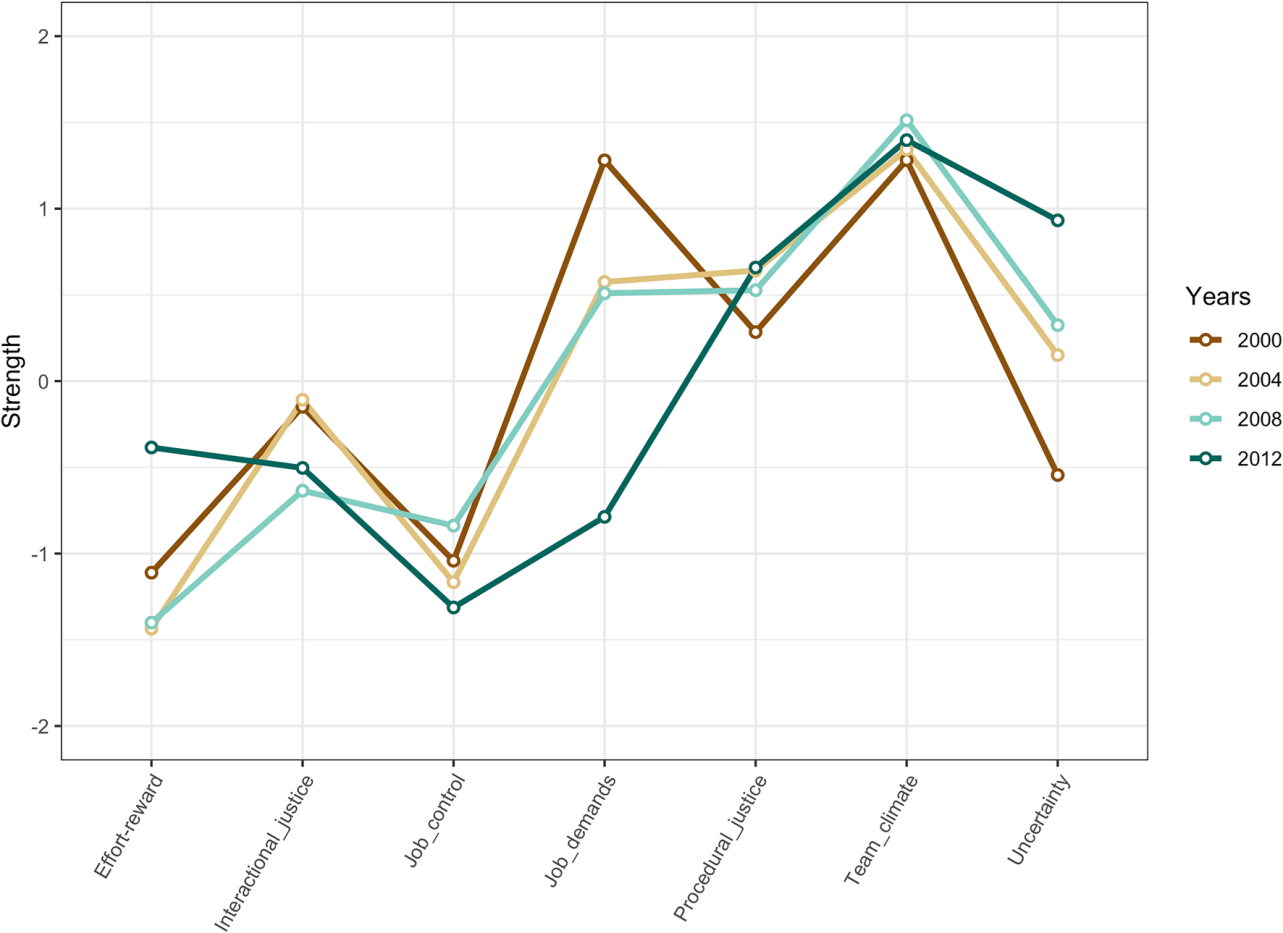
Strength centrality index for years 2000, 2004, 2008 and 2012.

The cross-sectional networks in each measurement point are presented in Fig. 1. Blue lines represent positive and red (dotted) lines negative partial correlations (a thicker edge denotes a larger correlation between two nodes). The sizes of the grey pies represent the explained variance in each psychosocial factor. The network layout was defined as the average layout (, but all networks were relatively similar. In all networks, procedural justice, interactional justice, and positive team climate were strongly associated with each other, as well as team climate and job control. Similarly, job uncertainty, high demands and effort-reward imbalance were associated with each other. Effort-reward imbalance was associated with low job control and inversely with both procedural and interactional justice. Job control and demands were also associated with each other. These associations were the pairwise associations between the mean levels of the psychosocial factors in each measurement point, when adjusting for the mean levels of all other psychosocial factor in the network. The most central psychosocial factor in the networks in all measurement points was team climate (Fig. 2). The correlations between centrality measures across different measurement points ranged from 0.40 (between 2000 and 2012) to 0.96 (between 2004 and 2008). The biggest differences in centrality measures between networks in different time points were found for job demands and uncertainty, potentially reflecting differences in the social and economic situations in different time points. Perceived uncertainty had in fact increased between 2000 and 2012 but the level of experienced job demands was relatively stable during the same time-period (Supplement Fig. S1). The correlation stability for strength centrality ranged from 0.672 to 0.750, meaning high stability. Values greater than 0.5 were considered stable.

**Figure 3:**
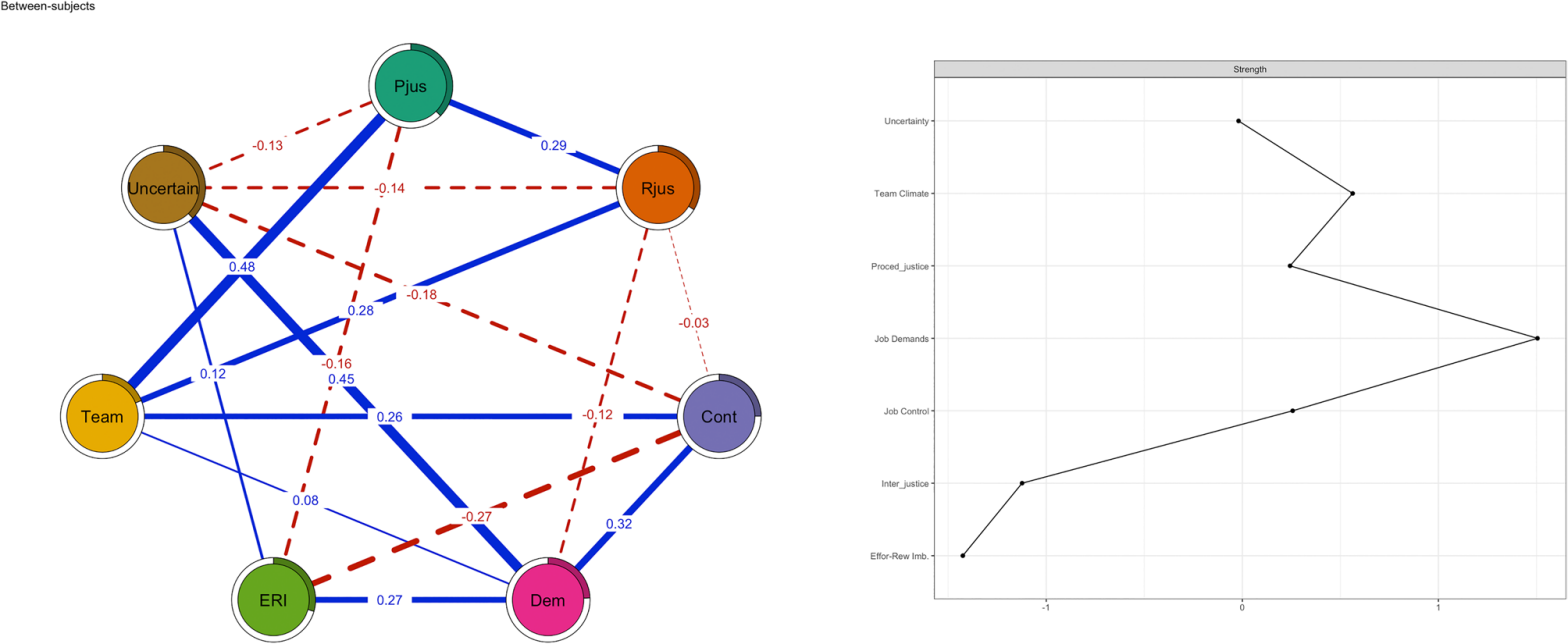
Between-individual longitudinal network (left panel) and strength (right panel) (procedural justice = pjus, interactional justice = rjus, effort-reward imbalance = eri, job demands = dem, job control = cont, uncertainty = uncer, team climate = team).

A between-individual network based on the longitudinal data is presented in Fig. 3. The between-individual network is comparable to cross-sectional networks, showing the pairwise associations among the mean levels of psychosocial factors across all measurement points after adjusting for the mean levels of all other psychosocial factors in the network. Blue lines represent positive and red (dotted) lines negative associations. The thickness of the line again corresponds to the strength of the association. The panels in the right-hand side show the centrality measures and the sizes of the pies represent the explained variances. In general, a positive connection between procedural justice, interactional justice, job control and team climate in this analysis indicated that during follow-up, people with higher scores on procedural justice tended to have higher scores on team climate and vice versa. Thus, the results were very similar to those presented in Fig. 1. The most central psychosocial factors in the between-individual network were job demands and team climate whereas the effort-reward imbalance was the least central.

**Figure 4:**
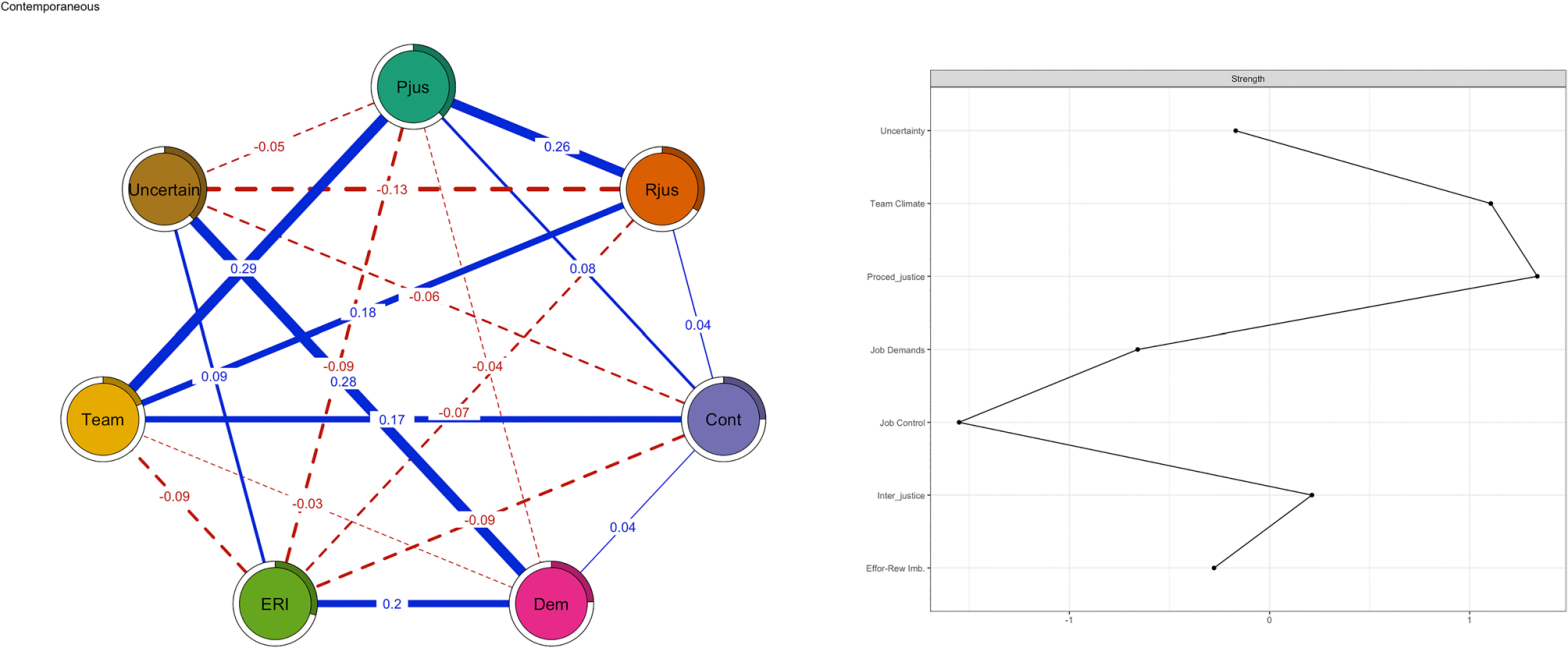
Contemporaneous relations between psychosocial factors (procedural justice = pjus, interactional justice = rjus, effort-reward imbalance = eri, job demands = dem, job control = cont, uncertainty = uncer, team climate = team).

The contemporaneous network (Fig. 4), including data from all measurement points of each psychosocial factor, illustrates the concurrent associations between the psychosocial factors. These associations are adjusted for temporal (longitudinal) effects and all other psychosocial factors at the same timepoint to compute a partial (independent of other associations) correlations network and thus take the temporal relationship into account. The relationships within each measurement point can be separately analyzed from relationships between measurement points in temporal models. In other words, it shows how psychosocial factors tend to co-occur at the same moment, controlling for all other psychosocial factors at the same moment and for all temporal relations among psychosocial factors. Similarly to the cross-sectional and between-individual networks, in the contemporaneous networks, procedural justice, interactional justice, team climate and job control were closely connected, as well as effort-reward imbalance, uncertainty and job demands. Job control was associated positively with favorable team climate and negatively with effort-reward imbalance. Effort-reward imbalance was negatively associated with both justice variables, team climate and job control. The most central psychosocial factors in contemporaneous network were team climate and procedural and interactional justice.

**Figure 5:**
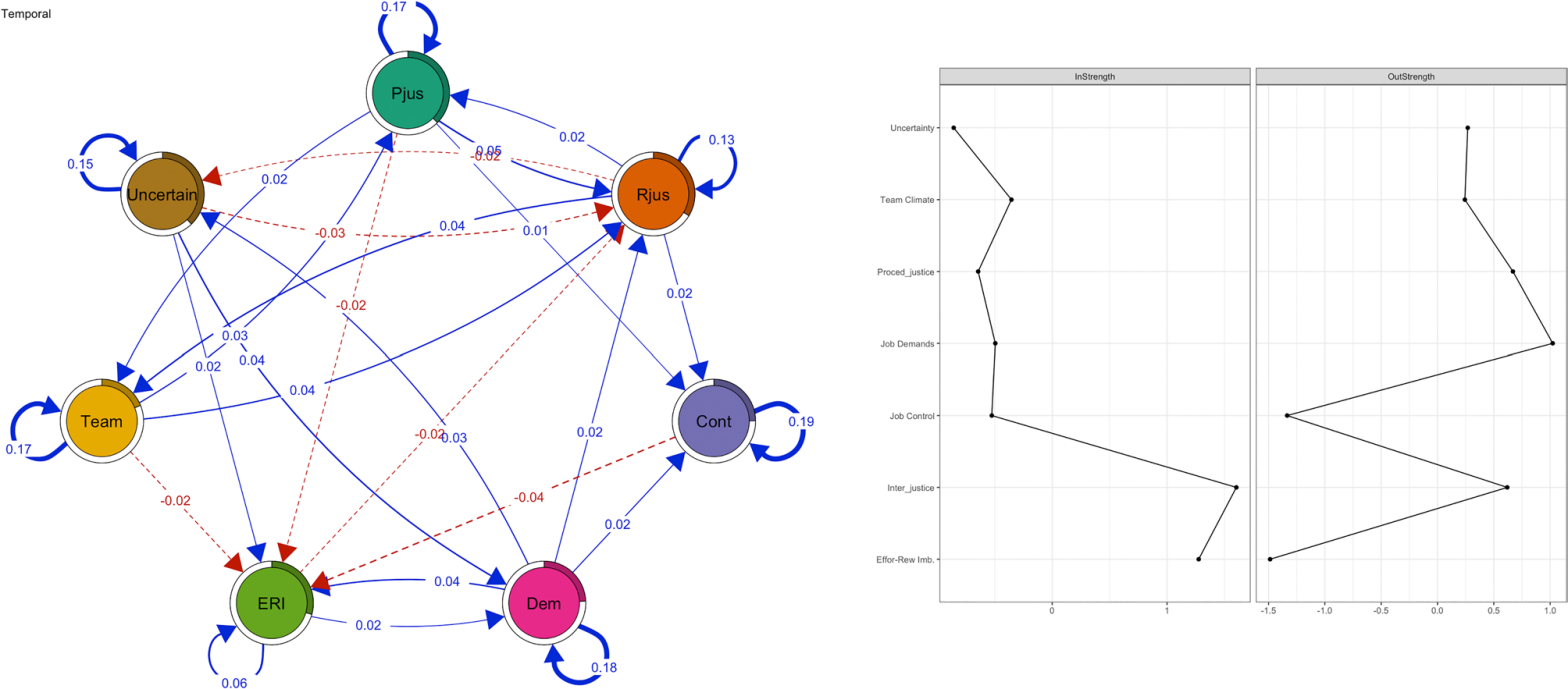
The temporal network between psychosocial factors (procedural justice = pjus, interactional justice = rjus, effort-reward imbalance = eri, job demands = dem, job control = cont, uncertainty = uncer, team climate = team).

The temporal network (Fig. 5) included the lagged directed associations based on longitudinal panel data. Each psychosocial factor at a certain measurement point was predicted by the same psychosocial factor and all other psychosocial factors in the previous measurement point, which provides information about the temporal multivariate relations. The left top panel in Fig. 5 shows how the lagged psychosocial factors predicted themselves (autoregressions) and each other across the follow-up while adjusting for all other lagged psychosocial factors. There were moderate to strong autoregressive loops with respect to all psychosocial factors across the measurement points. Not all associations in the temporal network were bidirectional. Based on centrality analyses, interactional justice and effort-reward imbalance seemed to be factors that were predicted by other psychosocial factors (high in-Strength), while job control and effort-reward imbalance appeared not to predict many other factors (low out-Strength). Job demands, and interactional and procedural justice, in turn, were the strongest predictors of other psychosocial factors in the network (high out-Strength).

## Discussion

In this study, we utilized network analysis to examine the interconnectedness of several work-related psychosocial risks and resources. We examined the structures of cross-sectional, concurrent, and temporal networks of the work-related psychosocial factors, and evaluated which factors are most central in the overall constellation of the estimated networks. Our analysis was based on several aspects of the psychosocial work environment derived from multiple complementary conceptual models: the Job Strain Model, the Effort-Reward Imbalance Model, the Team Climate Model, the Organizational Justice Model and the Job Uncertainty Model^7,12,20,24,44–46^.

As expected, the cross-sectional and contemporaneous networks showed a clear pattern of clustering (closely connected) of psychosocial factors that may be understood as general resources in the organization. These clustered factors were procedural justice, interactional justice, team climate and job control. Similarly, the adverse characteristics or risk factors, including effort-reward imbalance and uncertainty, were also clustered. Job control was inversely related to effort-reward imbalance which, in turn, was inversely associated with the justice perceptions and team climate. Greatest strength estimates were observed for team climate, and procedural and interactional justice, indicating these psychosocial factors were most connected with other psychosocial factors included in the cross-sectional and contemporaneous networks. These “clusters” may be understood also as partly overlapping constructs. Especially interactional justice and team climate may share some theoretical overlap as both are about the quality aspects of social relations within work environment. Similarly, ERI and uncertainty are both about the experienced status within ones organization.

In the longitudinal analysis with lagged and autoregressive associations, we observed relatively strong autoregressive loops with respect to all psychosocial factors over the follow-up. Not all associations between the different psychosocial factors were bidirectional, and there were differences in in-Strength and out-Strength centralities of the individual factors. Interactional justice and effort-reward imbalance were mostly predicted by other psychosocial factors (high in-Strength), and of these two factors, effort-reward imbalance did not predict many other psychosocial factors (low out-Strength). In contrast, job demands, interactional and procedural justice had high out-Strength estimates, suggesting they were central in predicting other psychosocial factors in the network. Similarly, job uncertainty had a relatively high out-Strength, but the lowest in-Strength estimate, suggesting that while uncertainty was relevant in predicting other psychosocial factors, these factors did not predict uncertainty. In sum, the longitudinal analyses showed clustering of resources and risk factors and suggested that interactional and procedural justice as well as job demands might be potentially efficient targets of interventions; improving these central ‘upstream’ factors could result in a spread of positive influences also in the other parts of the network. In contrast, effort-reward imbalance and, to lesser extent, job uncertainty, appeared as an endpoint rather than starting point in the chain of temporal associations between the psychosocial work characteristics.

The network approach is useful in health psychology and occupational medicine not only because it builds on recent statistical developments^59–61^, but also because it represents a fundamental shift in thinking about psychological well-being and health risks. As such, the network approach challenges the classical view postulating that health problems and their correlates arise from a common cause/disease (Common Cause -hypothesis)^27,62,63^. According to the recently developed Network Spread hypothesis, health problems develop with the spread of symptoms in causal networks^27^. Hence, the symptoms of depression, for example, may activate (or cause) each other in a certain pattern: insomnia may cause fatigue that, in turn, leads to concentration problems that may result in negative perceptions of oneself, and so forth. Moreover, the network approach views some of these patterns as more central than others in the networks of interrelated risks, potentially leading to more severe outcomes, such psychoses or major depressive episodes^64^.

The network analyses with multilevel vector autoregression (VAR) techniques have extended the more traditional multilevel analysis by allowing estimation of lagged associations between multiple variables as they change over time and facilitate evaluating hypotheses about time-dependent dynamic processes. Thus, network analyses can guide researchers towards more complex and dynamic thinking about psychosocial systems^63^. As far as we know, there are no studies focusing on the dynamics of work-related psychosocial factors using longitudinal network approach, although some studies have modelled psychiatric problems, including suicidality and auditory verbal hallucinations^58^ using VAR techniques. All the analyses could have been done using structural equation modeling (SEM) approach, and as have shown by Epskamp and others^66^, the results may be mathematically equivalent. However, for mainly exploratory search, as conducted in this paper, the search algorithms of VAR techniques are more efficient compared to building and testing various SEM model.

The study participants were Finnish workers from a female-dominated public sector, where working conditions, including psychosocial factors, could be relatively favourable in comparison to other sectors or countries. In industries or countries with long working hours or high work intensity, the associations between various psychosocial factors are likely different. The interpretation of the results of this study should thus be restricted to workplaces and societies with comparable working conditions. Although the Finnish public sector comprises a wide variety of occupational groups and socioeconomic levels, it is not known whether these networks of work-related psychosocial factors can be generalized to other occupational settings. Further studies are needed to evaluate the network structures of psychosocial work characteristics across various occupational groups and work environments.

## Conclusions

It has been argued that interventions aimed at improving psychosocial work environments target multiple factors in a blunderbuss fashion without a clear idea what the useful starting points would be^67^. Given that such strategies are inevitably inefficient, we sought to develop understanding of the network dynamics of several psychosocial work characteristics and thus contribute evidence on the potentially relevant starting points of efficient interventions. More evidence on the architecture and dynamics of work-related psychosocial factors could allow informed decisions about on whom, when, and how to intervene to improve work environments and work-related wellbeing.

## Methods

### Study sample

We obtained data from the Finnish Public Sector study which is a prospective cohort study among employees in the service of 10 municipalities and 5 hospital districts around Finland. These employees cover a wide range of socioeconomic levels from city mayors to semiskilled cleaners. The largest occupational groups in our data were nurses and teachers. In this study, we used survey data from four study phases 2000, 2004, 2008 and 2012. In the baseline sample, there were 9281 women and 1611 men (mean age 45 years, SD 8 years) and 17% had a university degree. Women were slightly overrepresented among the respondents (85% women) compared to eligible employees (76% women), but the differences in mean age (45 vs 44 years) and socioeconomic position (15% vs 17% performing manual labor) were small. Distributions of sex and age among respondents were close to the distributions among Finnish public sector employees (77% women; mean age 45 years)^25^. However, the predominance of women did not correspond to the gender distribution of the Finnish general working population (48% female; mean age 46 years)^68^.

The study was approved by the Ethic committee of the Hospital District of Helsinki and Uusimaa. All participants provided a written informed consent. This study was conducted according to the guidelines of the Helsinki declaration.

### Measures

*Job control* was measured using the Decision latitude (job control) scale derived from the Karasek’s Job Content Questionnaire (JCQ)^7,12^. The job control scale taps two concepts, *skill discretion* (the opportunities of an individual to develop his or her special abilities within the job, six items) and *decision authority* (individual’s abilities to be part of the decision-making process within the organization, three items). These subscales were combined for the analysis. Responses were given along a five-point scale from 5 = strongly agree to 1 = strongly disagree. The Cronbach’s α of the job control scales in ranged from 0.81 to 0.82 across the measurement points.

*Job demands* were measured with a workload scale developed by the Finnish Institute of Occupational Health^69^, which corresponds to demands in the Demand-Control model and has been linked to health outcomes in previous studies among hospital personnel^69^. The job demands scale consisted of four items, which considered time pressures and deadlines, lack of time to do what was expected, shortage of essential resources, as well as work overload. The respondents were asked to rate how often these stressors caused pressures and distress (from 1 = seldom or never to 5 = very often or continuously). The Cronbach’s α 0.77 in all measurement points.

*Team climate* was measured using the short version^70^ of the Team Climate Inventory (TCI)^71^. TCI conceptualizes team climate through four dimensions. *Participation safety* (four items) relates to active participation in work group interactions, where the interpersonal atmosphere is supportive and non-threatening. *Support for innovation* (three items refer to a climate where the attempts to introduce new and improved ways to do things in the work environment are anticipated, approved, and practically supported. *Vision* (four items) refers to the idea of a valued outcome in the workplace, how clearly defined and understandable the vision is, to which extent it is shared and accepted, and how attainable it is. *Task orientation* (three items) involves a general commitment to excellence in task performance and refers to a climate, which supports the adoption of improvements. Responses were given on a five-point scale (from 5 = strongly agree to 1 = strongly disagree). For the analysis, the subscales were combined (Cronbach’s α ranged from 0.91 to 0.92 during follow-up).

Perceived job *uncertainty* was assessed with four items requesting the extent to which the respondents’ work includes the following insecurity threats: (1) notice, (2) lay-offs, (3) redundancy, and (4) transfer to other jobs^72^. The items were rated on a five-point Likert scale ranging from 1 (very little) to 5 (very much). Cronbach’s α range was from 0.51 to 0.55.

*Effort Reward Imbalance* was measured using four survey questions [one on efforts and three on rewards] adapted from the standard 10-item ERI scale developed by Siegrist^73^. The response format was a 5-point Likert scale (1 = strongly disagree, 2 = disagree, 3 = neither agree nor disagree, 4 = agree, and 5 = strongly agree) and greater values indicated greater effort or rewards. The ratio of the self-assessed effort score and the mean of the self-assessed reward scores formed the individual-level ERI (i.e., individual level ERI = self-assessed effort/mean of self-assessed rewards). A higher ratio score indicated greater individual-level ERI. Cronbach’s α of the three effort items across the measurement points ranged from 0.64 to 0.68.

*Procedural justice* scale (seven items)^74^, considers whether the decision-making procedures at the workplace are accurate, correctable, consistently applied, and whether the procedures include opinions from the people involved. Cronbach’s α ranged from 0.92 to 0.93 during follow-up. Response format was a five-point scale (from 5 = strongly agree to 1 = strongly disagree).

*Interactional justice* scale (six items)^74^, assesses the quality of treatment employees experience in their interpersonal interactions during the completion of organizational processes (Bies, 2001). The scale includes items, such as whether the supervisors use kindness and consideration, are truthful, and can suppress personal biases. The response format was a five-point scale (5 = strongly agree to 1 = strongly disagree). For this scale, Cronbach’s α ranged from 0.91 to 0.92 during the follow-up.

As most of the psychosocial factors measured during the follow-up were not normally distributed (Supplement Table S1), we performed a nonparanormal transformation to all the variables using the Huge -package.

### Statistical analysis

The interconnectedness of seven higher-order work-related psychosocial factors—job control, job demands, team climate, uncertainty, effort-reward imbalance, procedural justice, and interactional justice—were examined using network analyses. The networks of work-related psychosocial factors were examined in four steps: first, we used a graphical Gaussian model (GGM) to estimate the basic *cross-sectional networks* at each measurement point. In these networks, *edges* represent the conditionally independent relationships between *nodes* when controlling for all other nodes^75^. We regularized the GGMs via the graphical LASSO (Least Absolute Shrinkage and Selection Operator) in order to shrink trivially small associations to zero (remove “false positive” edges)^76^. We used the R package “qgraph”^77^ that implements the graphical LASSO regularization in combination with extended Bayesian information criterion (EBIC) model selection and we set the value of the hyper-parameter gamma (γ) to 0.5 to minimize the probability of including false positive edges^75^.

Second, we assessed the *predictability* of each individual (higher-level) psychosocial factor, that is, how much of the variance of each psychosocial factor (node) is explained by the other psychosocial factors (nodes) in the network, using the R package MGM, which was developed for estimating time-varying mixed graphical models^78^ version 1.1.-7. The MGMs were also estimated via 1-regularized (LASSO) neighborhood regression, where neighborhood of a node refers to the set of nodes connected to it.

Third, we computed *centrality indices* to quantify the importance of each psychosocial factor (node) in the network^79^. Node strength centrality measures the weighted number of connections of a focal node and thereby the degree to which it is involved in the network.

Fourth, we used multilevel vector autoregression (VAR) techniques (for panel data), in which a variable at a certain time point (t) is predicted by the same variable at the previous time point (t − 1) (autoregressive effects) and all other variables at t − 1 (lagged effects) Here, the package Psychonetrics^80^ was used to detect *longitudinal association*s between psychosocial factors. The autoregressive and lagged effects were quantified and visualized in a network. Epskamp et al.^80^ introduced an additional network for estimating EMA data (a contemporaneous network) that can be used in the partial association between the residuals of the temporal network that are the result of associations between the variables that are not explained by the current chosen time interval, the chosen lag, or anything else that is not explicitly measured and modeled. In the contemporaneous network, the edges between nodes represent the partial correlation obtained after controlling for both temporal effects and all other variables in the same window of measurement^80,81^. When data are collected from multiple subjects, between-person networks using EMA data may also be estimated. Between-subject predictors are calculated using the covariance structure of stationary means. We also calculated the in-Strength and out-Strength centrality indices for the temporal network to identify factors that were the strongest predictors of other factors and the factors that were mostly predicted by other factors.

We tested the robustness of the cross-sectional networks and derived from them using the R package “bootnet”, a bootstrap sampling procedure (nBoots = 1000)^82^. We evaluated the stability of the centrality metrics by repeatedly correlating the centrality metrics of the original data set with those calculated from subsamples including progressively fewer participants. We quantified the effects by calculating the centrality stability correlation coefficient (CS-coefficient). The CS-coefficient represents the maximum proportion of participants that can be dropped while maintaining 95% probability that the correlation between centrality metrics from the full data set and the subset data are at least 0.70.

All analyses were conducted using R 3.6.1.

## Supplementary Information

Supplementary Figure 1.

Supplementary Table 1.

## Data Availability

The data that support the findings of this study are available from the Finnish Institute of Occupational Health, but restrictions apply to the availability of these data, which were used under license for the current study, and so are not publicly available. Data are however available from the authors upon reasonable request and with permission of the Finnish Institute of Occupational Health.
