# Supplementary figures and images for: Psychosocial work environment as a dynamic network: a multi-wave cohort study
Source: Sci Rep. 2022 Jul 28;12:12982. doi: 10.1038/s41598-022-17283-z (PMC9334355; doi:10.1038/s41598-022-17283-z)

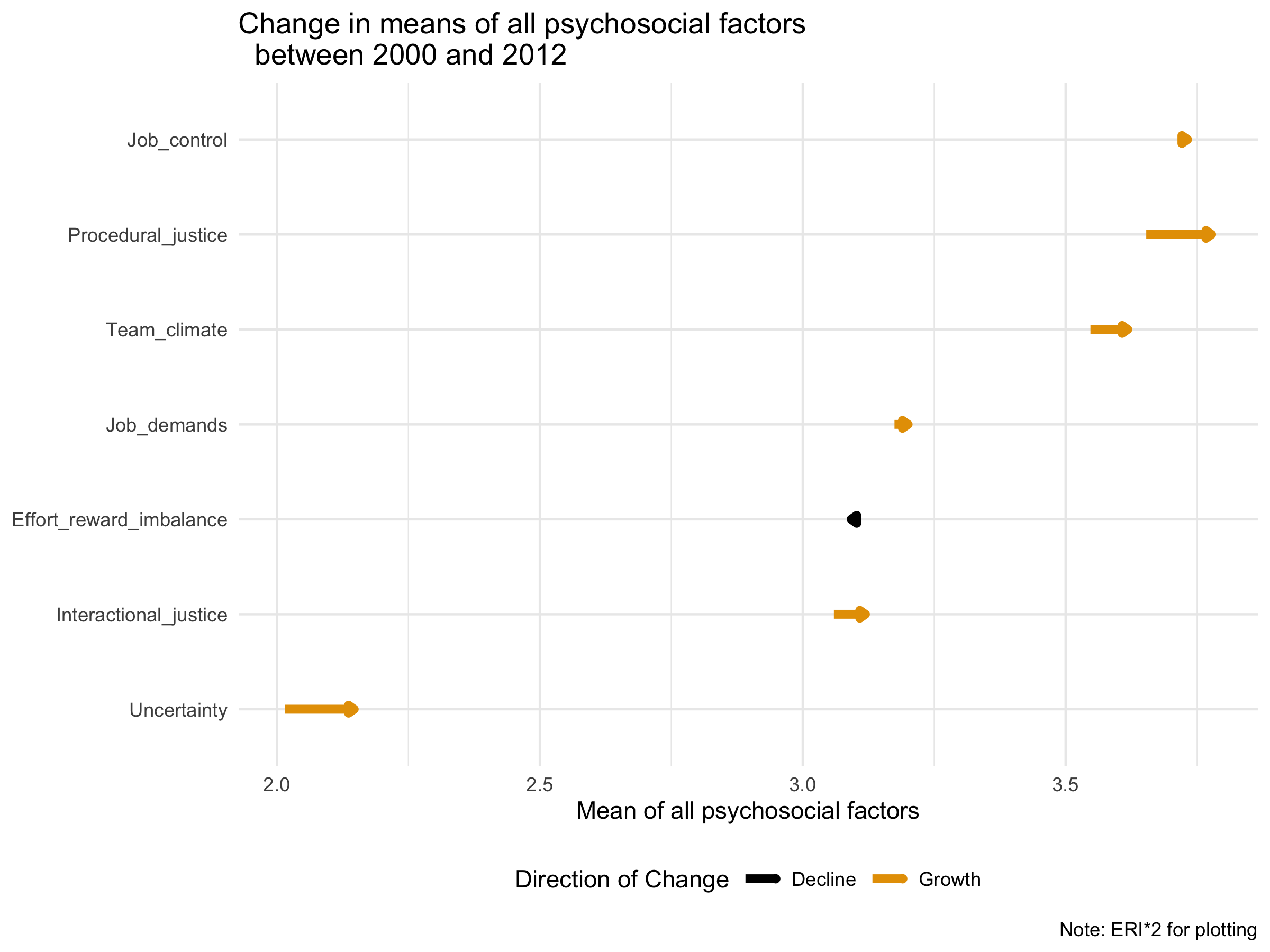

Supplement: Supplementary file 1 — Supplementary Figure 1. [file 41598_2022_17283_MOESM1_ESM.png]
