# Supplementary material for: Psychosocial work environment as a dynamic network: a multi-wave cohort study
Source: Sci Rep. 2022 Jul 28;12:12982. doi: 10.1038/s41598-022-17283-z (PMC9334355; doi:10.1038/s41598-022-17283-z)
Supplement: Supplementary file 2 — Supplementary Table 1. [file 41598_2022_17283_MOESM2_ESM.docx]

| STable 1. Normality tests in all variables | | | |
| --- | --- | --- | --- |
| **Items** | **skew** | **Z** | **p-value** |
| pjustice_2000 | -0.61 | -22.3 | <.000 |
| pjustice_2004 | -0.65 | -23.47 | <.000 |
| pjustice_2008 | -0.7 | -25.17 | <.000 |
| pjustice_2012 | -0.75 | -26.55 | <.000 |
| rjustice_2000 | -0.01 | -0.55 | 0.583 |
| rjustice_2004 | 0.01 | 0.33 | 0.742 |
| rjustice_2008 | -0.04 | -1.64 | 0.101 |
| rjustice_2012 | -0.1 | -3.87 | <.000 |
| eri_2000 | 2.09 | 53.95 | <.000 |
| eri_2004 | 2.27 | 56.4 | <.000 |
| eri_2008 | 2.36 | 57.61 | <.000 |
| eri_2012 | 2.41 | 58.21 | <.000 |
| jdemand_2000 | -0.01 | -0.3 | 0.765 |
| jdemand_2004 | 0 | 0.1 | 0.918 |
| jdemand_2008 | 0.02 | 0.61 | 0.543 |
| jdemand_2012 | 0 | -0.17 | 0.866 |
| uncertain_2000 | 0.83 | 28.85 | <.000 |
| uncertain_2004 | 0.67 | 24.19 | <.000 |
| uncertain_2008 | 0.67 | 24.18 | <.000 |
| uncertain_2012 | 0.69 | 24.88 | <.000 |
| jcontrol_2000 | -0.57 | -21.04 | <.000 |
| jcontrol_2004 | -0.56 | -20.53 | <.000 |
| jcontrol_2008 | -0.59 | -21.69 | <.000 |
| jcontrol_2012 | -0.54 | -20.19 | <.000 |
| team_2000 | -0.47 | -17.72 | <.000 |
| team_2004 | -0.43 | -16.24 | <.000 |
| team_2008 | -0.51 | -18.89 | <.000 |
| team_2012 | -0.52 | -19.5 | <.000 |
